# Primary glioblastoma multiform (GBM) of the optic nerve and chiasma: A case report and systematic review of the literature

**DOI:** 10.1002/ccr3.8636

**Published:** 2024-03-20

**Authors:** Ali Mulhem

**Affiliations:** ^1^ Department of Neurosurgery Vivantes Klinikum im Friedrichshain Berlin Germany; ^2^ Department of Continuing Education, MSc Program in Evidence‐Based Health Care University of Oxford Oxford UK

**Keywords:** case report, chiasma, glioblastoma, optic nerve, systematic review

## Abstract

**Key Clinical Message:**

Primary GBM of the optic nerve and chiasma should be included in the differential diagnosis of progressive lesions despite initial treatment; clinicians should avoid delay in confirming the histology to initiate proper treatment and improve prognosis.

**Abstract:**

Primary GBM of the optic nerve or chiasma is very rare. The characteristics of this condition have not been well‐described, which poses difficulties in establishing the correct diagnosis, affecting the treatment and the prognosis. We present a case of GBM of the optic chiasma diagnosed through an open biopsy at our centre. Following the PRISMA statement, we also conducted a systematic review after protocol registration in PROSPERO (CRD42021285855). We searched Medline and Embase through Ovid from inception until December 31, 2021. Two reviewers independently screened the studies. Studies were eligible for inclusion if they reported cases of primary GBM confined to the optic nerve or chiasma as the initial radiological diagnosis. A 77‐year‐old female was referred for progressive visual loss lasting 8 weeks. MRI revealed a suspected lesion in the left chiasma. The patient's vision deteriorated further despite initially diagnosing an inflammatory process and empirical treatment with corticosteroids. Subsequently, the patient underwent an open biopsy and surgical debulking. Histology, including epigenetic analysis, confirmed GBM grade IV. Radiochemotherapy was administered. The patient died 19 months after surgery. We identified 45 similar cases (22 female) reported in 35 studies between 1949 and 2020. The mean age of the cases was 61 (SD = 14.6). Most cases were misdiagnosed and mistreated accordingly, so there was a median delay of 8 weeks (IQR: 5–14 weeks) in obtaining histological confirmation of the diagnosis, delaying the initiation of appropriate treatment. Five cases became no treatment since the patients died shortly after the delayed histologic diagnosis. The Kaplan–Meier estimate indicated that most patients died within 20 months of presentation, with a 1‐year survival rate of 50%, and untreated cases had very low survival rates compared to treated cases. Primary GBM of the optic nerve and chiasma is a rare condition primarily affecting adults. The rarity of this condition contributes to initial misdiagnosis, mistreatment, and delays in confirming the histology and initiating appropriate treatment. The prognosis remains poor, but treatment, including surgery and radiochemotherapy, improves survival.

## 1 BACKGROUND

Primary GBM of the optic nerve or chiasma is a rare condition. It refers to GBMs that initially originate from the optic nerve or chiasma as malignant tumors (WHO grade IV) and can later progress to other anatomical regions. It is important to distinguish them from secondary GBM, which develops from lower‐grade gliomas (WHO grade I, II, or III) and is more common, as well as from secondary progression of primary GBM originating from other anatomical regions such as the hypothalamus, frontal, or temporal lobes. While there have been isolated reports of primary GBM confined to the optic nerve since the mid‐20th century, the first comprehensive description of this malignant tumor as an independent entity in this anatomical region was in 1973 by William Hoyt.^1^ He reported five cases of malignant glioma, one of which was primary GBM in adults, and distinguished them as a separate oncological entity from secondary GBM. Since then, sporadic cases have been published worldwide to provide a more detailed clinical understanding of primary GBM, including demographic features, presentation, diagnosis, treatment, and prognosis. However, due to the rarity of these tumors, this clinical understanding is still limited among clinicians. Additionally, establishing the diagnosis^2^ poses difficulties and often leads to delays in initiating appropriate treatment, which can impact outcomes. This study aims to present a case of primary GBM of the optic chiasma and systematically review the existing medical literature for similar published cases in this anatomical region to provide a more detailed understanding of this entity, its clinical course and the impact of delayed diagnosis.

## 2 METHODS

We present a case of GBM of the optic chiasma that was diagnosed and surgically treated at our centre. The patient's history, findings, imaging, treatment, and follow‐up were detailed. In the next phase, a systematic review was conducted following the PRISMA statement^3^ after protocol registration in PROSPERO (CRD42021285855). A search was performed through Ovid in Medline and Embase from inception until December 31, 2021. Two reviewers independently screened the studies using a two‐stage approach (title/abstract phase and full‐text phase) with the assistance of Covidence software.^4^ Studies were included if they reported cases of primary GBM limited to the optic nerve or chiasma in the initial radiological diagnosis. Exclusions were made for secondary GBM (transformation of lower‐grade glioma, gliosarcoma, and anaplastic glioma). Cases describing optic nerve or chiasma involvement due to extension and progression of primary GBM from neighboring regions (hypothalamus, hypophysis, third ventricle, etc.) were also excluded. The reviewers independently extracted the data into an Excel spreadsheet, and any discrepancies in screening and data extraction were resolved through discussion. The agreement between reviewers was measured using kappa statistics. Descriptive statistics for the included cases, including Kaplan–Meier estimates, have been presented.^5^

## 3 RESULTS

### 3.1 Case presentation

#### 3.1.1 History before initial presentation at our centre (Time 0)

**FIGURE 1:**
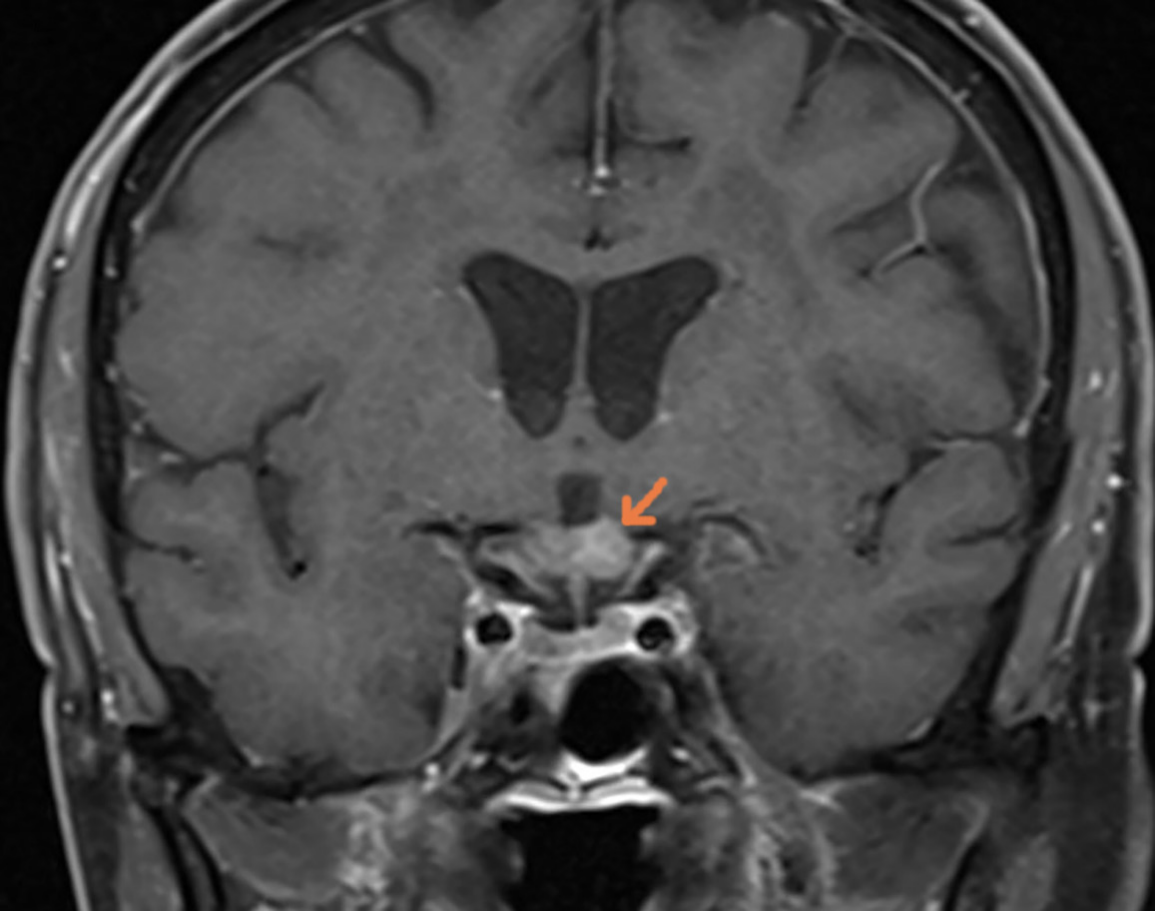
Preoperative MRI, coronal T1 with contrast, showing the lesion of the chiasma.

**FIGURE 2:**
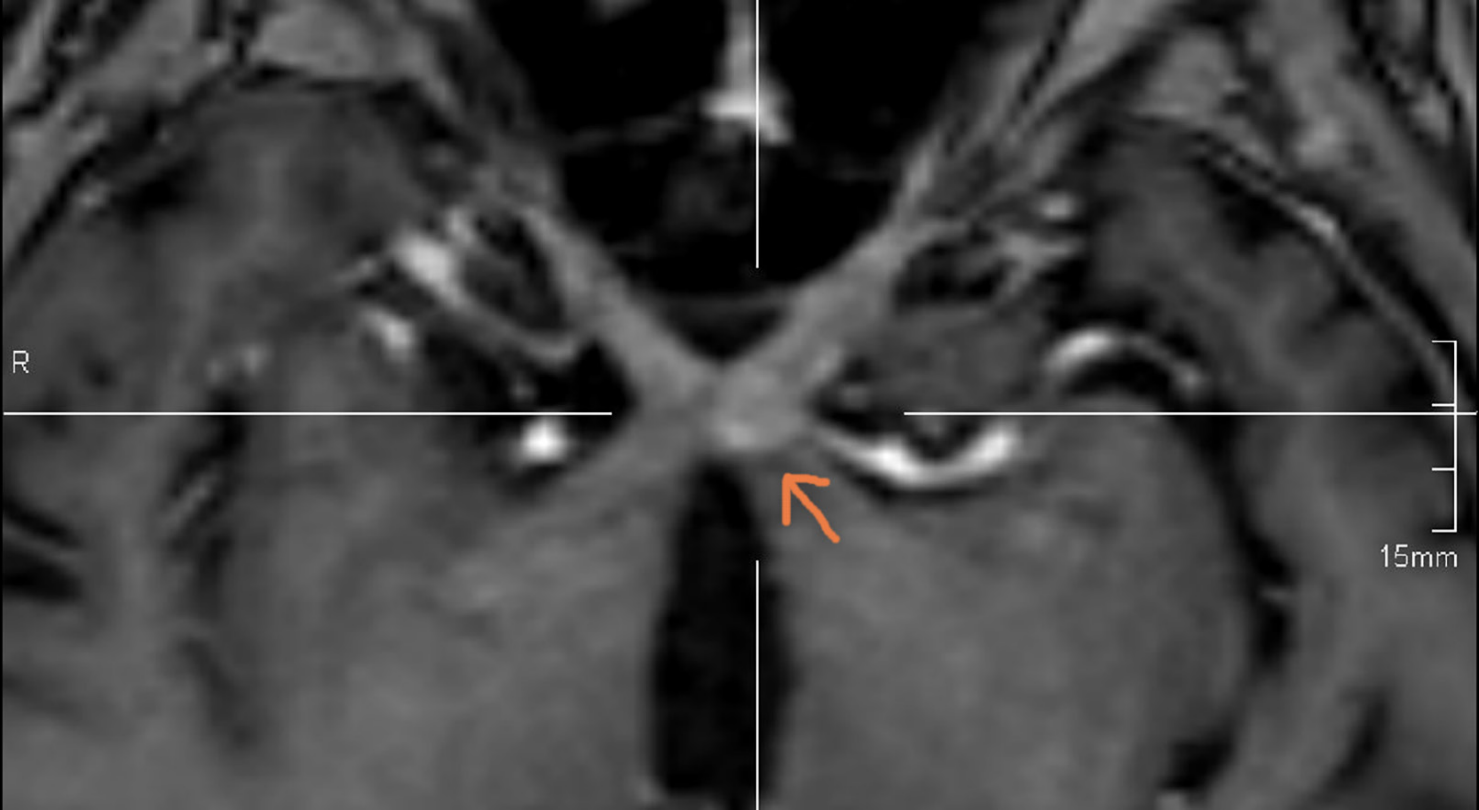
Preoperative MRI, axial T1 with contrast, showing the lesion of the chiasma.

A 77‐year‐old female with no prior medical or familial history was referred to our centre due to a progressive bilateral visual loss lasting more than 8 weeks. The left eye was affected initially 2 months ago, followed by the right eye 4 weeks later. The patient had previously sought care in three outpatient clinics without undergoing further diagnostic procedures. Two weeks later, the patient visited another centre's emergency room due to worsening symptoms and visual field disturbances (see Figure S1). CT scan showed a suspected lesion on the left chiasma (Figure S2), and the MRI in the T1‐sequence revealed a space‐occupying lesion in the left chiasma with contrast enhancement (Figures 1 and 2); in T1 sequence with no contrast the lesion appeared isointense (Figure S3) and relatively hyperintense in T2 sequence (Figure S4). Initially, the lesion was believed to be an inflammatory process and treated with dexamethasone alone.

#### 3.1.2 Initial presentation at our centre (Time 1)

Despite the corticosteroid treatment, the patient's visual acuity continued to deteriorate, prompting her to seek another opinion at our centre. The initial ophthalmological examination showed visual acuity 12/20 in the right eye and only light perception in the left eye, accompanied by visual field disturbances (right: quadrantanopia, left: solitary optotypes; see Figure S5). Due to the rapid deterioration observed between Time 0 and Time 1, lack of response to corticosteroid therapy, and negative laboratory results for systemic inflammatory diseases (such as sarcoidosis or toxoplasmosis), we began to suspect the nature of the lesion. Consequently, considering the exclusion of inflammatory processes, poor response to empirical corticosteroid therapy, and the radiological differential diagnosis of a mass lesion (see Figures 1 and 2), we proposed an open surgical biopsy to investigate the lesion further.

#### 3.1.3 The course of treatment (Time 2)

**FIGURE 3:**
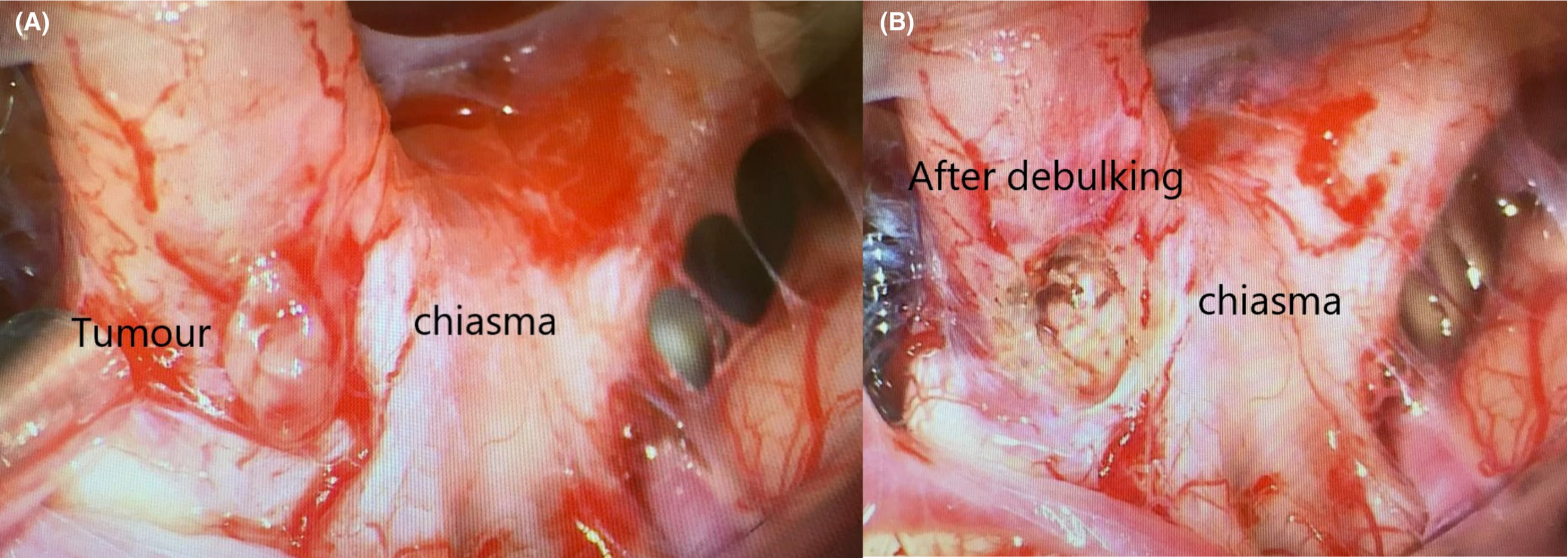
Intraoperative image, (A) showing the tumor arising from the chiasma, and (B) after taking the biopsy and performing debulking.

**FIGURE 4:**
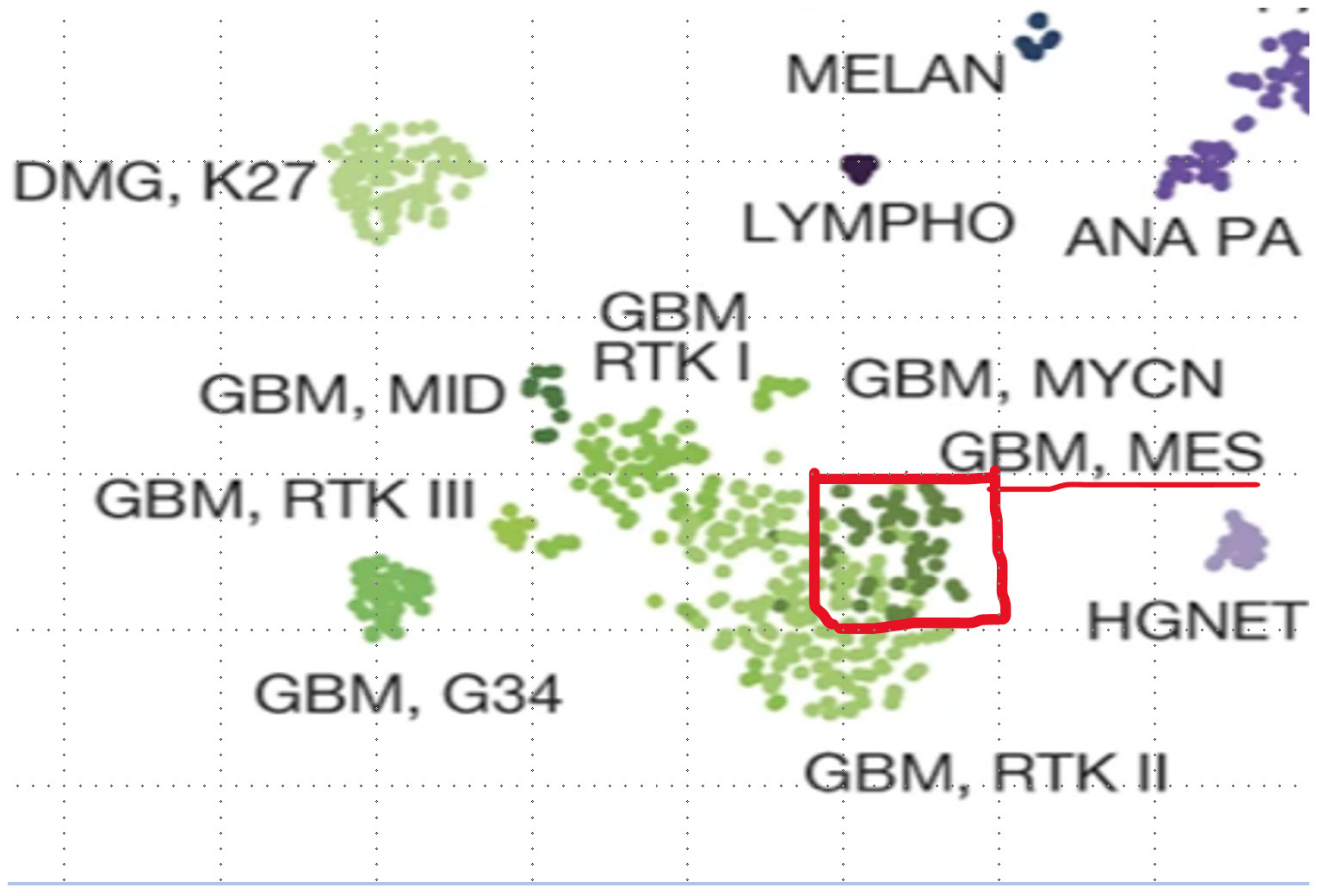
The epigenetic card of the analysis of the biopsy, corresponding with GBM grade 4.

The operation took place 5 days after the presentation in our centre, using a transcranial frontotemporal approach from the left side. Intraoperatively, the lesion was found to originate from the optic chiasma (Figure 3 left). Surgical debulking and biopsy were performed (Figure 3 right). Histological examination, including epic‐analysis, confirmed GBM grade IV, wild type (Figure 4). The vision was preserved in the right eye immediately after the surgery. Subsequently, the patient underwent radiochemotherapy with 44 Gy and Temozolomide (75 mg/m2/day), following the Stupp regimen.^6^

#### 3.1.4 Follow‐up findings (Time 3)

**FIGURE 5:**
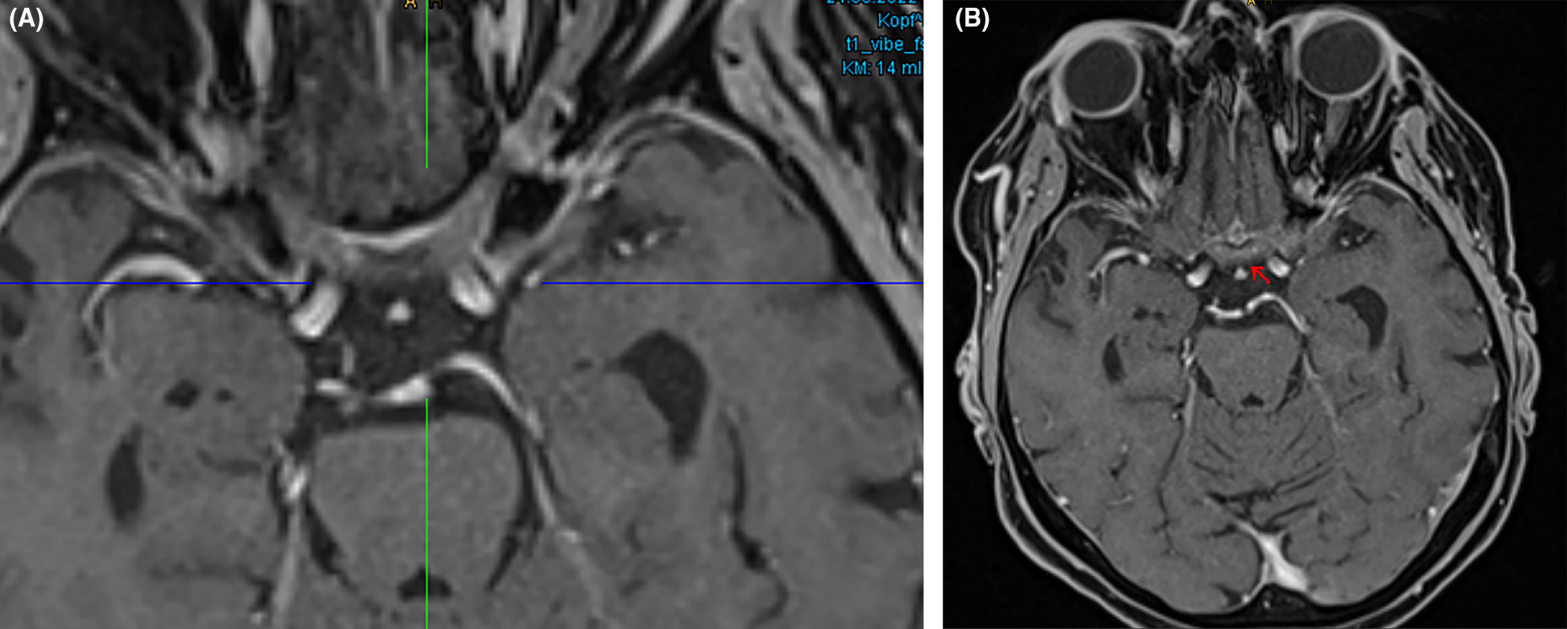
(A) Follow‐up MRI, axial T1 with contrast, showing no progress after tumor removal in the chiasma region. (B) Follow‐up MRI, axial T1 with contrast, showing no progress after tumor removal intracranially.

During the 18‐month follow‐up after the operation, the patient's overall clinical condition was generally good. However, she experienced complete vision loss in both eyes, which is probably attributed to the radiation (with 44 Gy) since the follow‐up MRI in the T1 sequence with contrast showed no suspected lesions or progression with no enhancement locally (Figure 5A) or intracranially (Figure 5B). The patient died 19 months after surgery because of hypernatremia caused by pituitary gland dysfunction, which we attributed to a delayed complication of the radiotherapy.

### 3.2 Literature review

**FIGURE 6:**
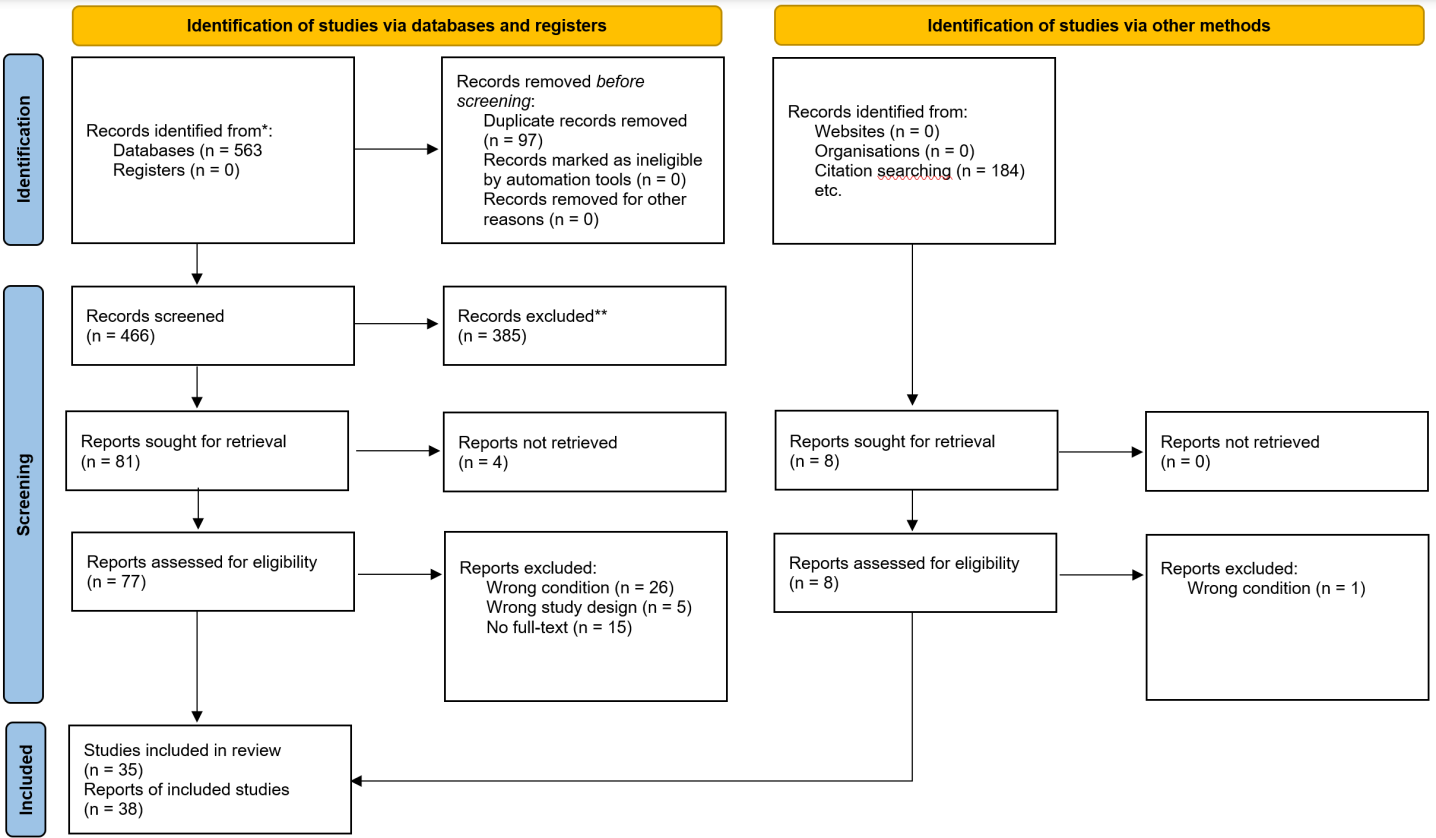
PRISMA flowchart of the systematic review.

We reviewed 747 publications, and after screening and assessing for eligibility, 35 studies (reported in 38 publications between 1949 and 2020) were included in the review (Figure 6). There was a good agreement in the screening and eligibility assessment, with a kappa value of 0.5 (SE = 0.05) for screening and 0.6 (SE = 0.09) for eligibility. The included studies reported 45 similar cases, resulting in a sample size 46, including ours. Table 1 provides a summary of all the included cases and their characteristics. The mean age of the entire sample was 61 years, with a standard deviation (SD) of 14.6. Of the 46 cases, 22 were female. Radiological confirmation was obtained through MRI in 33 cases since 1995, while the remaining cases were diagnosed using CT scans or autopsy. At the time of radiological diagnosis, 34% of the cases showed signs of optic tract involvement, and 12 cases exhibited primary involvement of the entire optic pathway (nerve, chiasma, and tract).

**TABLE 1:** Characterization of all reported cases of primary GBM of the optic nerve and chiasma^a^, ordered ascendingly according to year of publication.

First author, Year of publication	Age in years/Sex^b^	Clinical presentation	Radiological modality	Anatomical region^c^/side	Therapy^d^	Survival time in months
Sæbø (1949)^27^	43/F	Decrease in vision through 4 months and simultaneous protrusion of the left eye	–	N/left	R + S	12
Mattson (1966)^20^	59/F	Progressive blindness of the left eye in 1 week	X‐ray	N, C, T/left	No treatment	4
Hoyt (1973)^1^	55/M	Progressive painful blurring of vision in left eye	CT	N, C, T/left	R	9
Manor (1976)^21^	70/M	Progressive vision decrease and headaches	CT	N, C, T/both	No treatment	4
Harper (1978)^22^	75/M	2 months of bilateral blurting of vision	CT	N, C, T/both	No treatment	3
Spoor (1980)^28^, ^29^	64/F	Progressive bilateral visual dysfunction	CT	N, C/both	R	9
	60/F	3‐week history of rapidly decreasing vision and bifrontal headaches	CT	N/left	R	6
Barbaro (1982)^11^	26/M	Progressive visual loss and headache for 6 weeks	CT	N, C/both	R + C + S	8
Evens (1987)^30^	61/F	Bilateral vision loss	–	N, C/both	R	9
Albers (1988)^31^	51/M	–	–	−/right	R	20
	40/F	–	–	−/left	R + C	18
Woiciechowsky (1995)^32^	76/M	Progressive loss of vision	MRI	N, C, T/left	R + S	7
Pallini (1996)^33^	59/F	Progressive loss of vision and diabetes insipidus	‐	C/both	R	7
Cirak (2000)^34^	6/F	Progressive deterioration of vision in both eyes over 2 months	MRI	C/both	S	0,25
Cummings (2000)^35^	65/F	Visual signs and symptoms	MRI	N, C/−	R + S	12
Hahn (2004)^36^	53/M	Acute visual loss in the left eye within 1 day	MRI	N, C, T/left	R + C	‐
Danesh‐Meyer (2005)^12^	70/F	Blurred vision with 3 weeks deterioration right	MRI	N, C/right	R + C	11
Hartel (2006)^23^	59/M	Sudden vision loss	MRI	N, C/both	No treatment	2
Dinh (2007)^7^	48/F	Subacute onset of visual deterioration	MRI	C/left	R + S	13,75
Balachandran (2009)^13^	60/M	Difficulty vision in his left eye and headache	MRI	N, C, T/left	R + C	4
Matloob (2011)^14^	63/F	2‐day history of progressive visual loss in the right eye with retrobulbar pain	MRI	N, C/right	C	6
Kang (2012)^15^	60/F	1 week of progressive painless bilateral vision loss	MRI	N, C, T/both	R + C	8
Raper (2013)^19^	60/M	Decreased visual acuity in the right eye	MRI	N, C, T/right	–	–
	79/M	Visual field defect	MRI	C, T/left	R + C	–
Jiang (2013)^37^	67/F	Headaches, total vision loss in left eye, and partial in the right	MRI	N, C/both	–	5
	61/F	Blurred vision in her right eye	MRI	N, T/right	–	–
Ashur‐Fabian (2013)^8^	64/M	Right eye vision loss	MRI	N, C/right	R + C+ medically induced hypothyroidism	54
Kim (2014)^16^	54/M	Isolated, progressive visual loss for 6 weeks	MRI	N, C/left	No treatment	12,6
Caignard (2014)^38^	74/M	Progressive visual loss in left eye	MRI	N, C/left	R + C	15
	74/F	Bilateral visual loss	MRI	C, T/both	R + C	11
Pecen (2014)^17^	61/F	Acute onset of painless blurred vision in the right	MRI	N, C/right	R + C	10
Colpak (2014)^39^	47/M	Sudden visual loss in the left eye	MRI	N/left	No	2,75
Traber (2015)^25^	65/M	Left‐sided visual loss for 5 weeks	MRI	N, C/left	R + C	4,5
	54/M	Painful eye movements and right visual loss	MRI	N/right	R + C	18
	75/M	Painless unilateral visual blur, impaired color vision, and RAPD in the right eye	MRI	N/right	R	12
	76/F	Painless visual loss in the right eye for 5 weeks	MRI	N, C, T/both	R	7
Lyapichev (2016)^40^	82/M	Gradual vision loss of the left eye in 1 month	MRI	N, C/left	S	2
Shows (2016)^41^	56/M	–	–	N/right	–	1
Alireza (2017)^9^	49/M	Vision discomfort and decrease of visual acuity in both eyes	MRI	C/both	C	4
	67/F	Decrease of visual acuity of the right eye	MRI	N, C/right	R + C	30
	86/F	Rapid deterioration of visual acuity in both eyes	MRI	N/both	C	5
Mastorakos (2018)^10^	66/M	Right nasal hemifield visual deficit and reduced visual acuity in his right eye	MRI	N, C, T/right	R + C + S	16
Rizzo (2018)^24^	80/M	Slowly progressive bilateral visual loss	MRI	N, C, T/right	–	–
Kalnins (2019)^2^	72/F	Left orbital pain with eye movement and left inferior nasal quadrantanopia	MRI	N/left	R + C	15
Ramakrishnan (2020)^26^	48/M	Decreased vision and pain of the right eye	MRI	N/right	R + C	12
Present study	77/F	Progressive bilateral visual loss left>right	MRI	C/left	R + C + S	–

^a^ Missed data presented as (−).

^b^ F = Female, M = Male.

^c^ N = optic nerve, C = optic chiasma, T = optic tract.

^d^ R = Radiation, C = Chemotherapy, S = Surgery.

**FIGURE 7:**
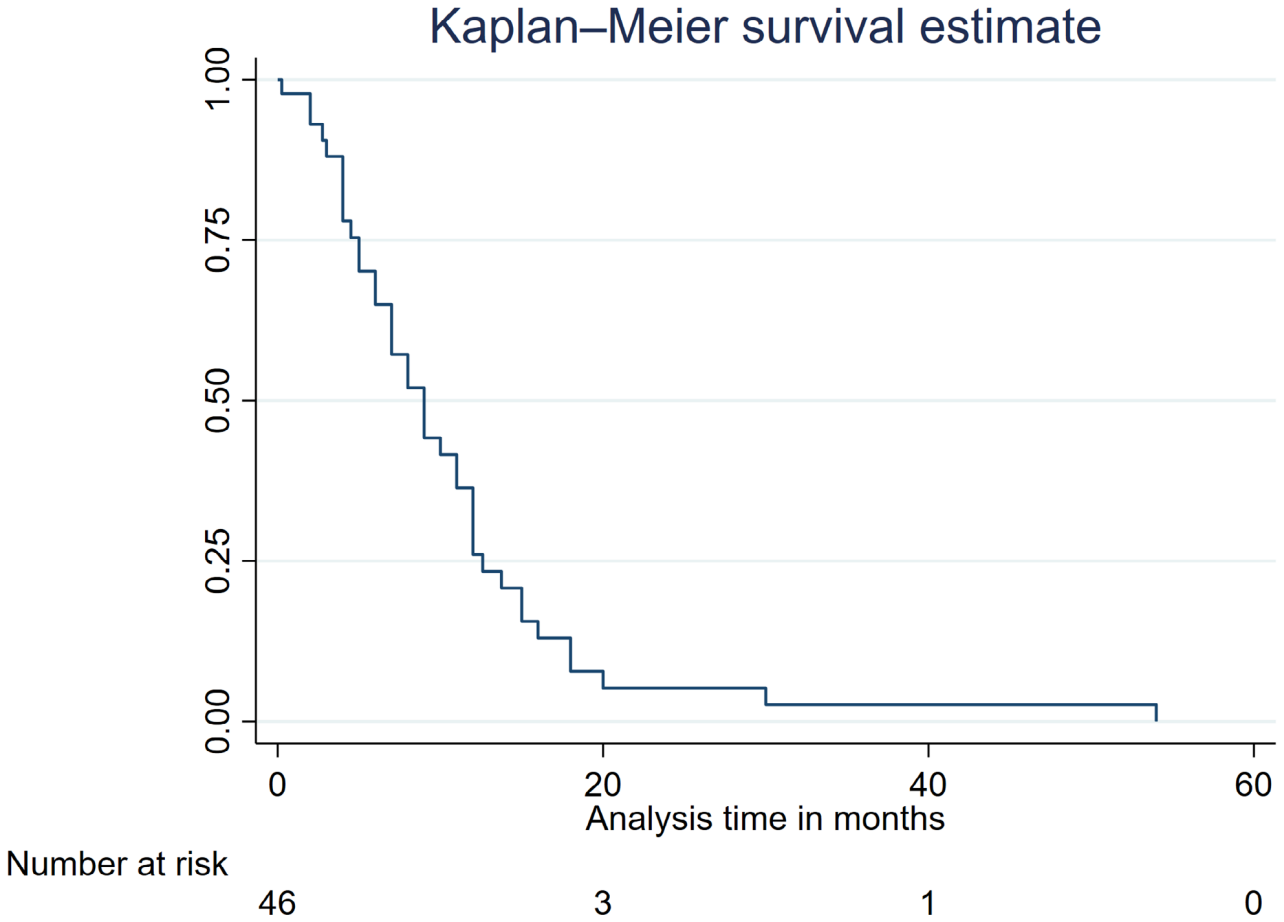
Kaplan–Meier estimate of the survival of all included cases.

Regarding lesion location, 39% were left‐sided, 29% were right‐sided, and 32% involved both sides. Histological confirmation of the diagnosis was often delayed, with a median delay of 8 weeks and an interquartile range (IQR) of 5–14 weeks. Six cases received no treatment, while others underwent surgery (partial resection in only nine cases), radiation therapy, chemotherapy, or a combination of treatments (Table 1). According to the Kaplan–Meier estimate, most patients died within 20 months of presentation, with a 1‐year survival rate of 50% (Figure 7). Only one untreated case achieved 1‐year survival (Kim, 2014), and the patient died 2 days after the biopsy, which has been delayed because of medical insurance problems. On the contrary, all other untreated cases had very low survival rates compared to treated cases (4 months in Mattson, 1966; 4 months in Manor, 1976; 3 months in Harper, 1978; 2 months in Hartel, 2006) (see Table 1). The specific treatment of GBM was not initiated in these cases because of the delay in confirming the histological diagnosis, so that the patients died before appropriate treatment.

## 4 DISCUSSION

Primary glioblastoma of the optic region is rare and difficult to diagnose. Over the last 70 years, there has been approximately one reported case every 2 years worldwide.^7^, ^8^, ^9^, ^10^ This condition primarily affects adults in their 50's and 60's, with the youngest reported case being 26.^11^ At presentation, patients exhibit clinical symptoms and signs that mimic other acute or subacute ophthalmic conditions, such as ischemic or venous congestive optic neuropathy or inflammatory disorders, making the diagnosis challenging.^2^, ^12^, ^13^, ^14^, ^15^, ^16^, ^17^, ^18^ Raper emphasized in his report of three cases that the clinical presentation of optic nerve GBM does not correlate with the anatomical changes of the optic nerve; typically, the fundoscopic examination is normal upon presentation (the patient cannot see, and the ophthalmologist cannot see).^19^ The only systematic review summarizing previous cases of primary GBM in the optic region was conducted in 2017. It included cases of anaplastic glioma and glioblastoma, limiting the inclusion to cases reported after 1973.^9^ This case report and literature review aim to characterize this oncological entity and emphasize the importance of histological confirmation for any progressive space‐occupying lesion in the optic nerve or chiasma that does not respond to empirical therapy and after other differential diagnoses have been excluded. Clinicians may be hesitant to perform invasive surgical biopsies due to the risk of neurological deterioration; however, the trade‐off of achieving an accurate diagnosis earlier is noteworthy since the lack of treatment for GBM leads to poor survival outcomes.^20^, ^21^, ^22^, ^23^

To date, 46 cases have been reported in the literature. These cases presented with gradual bilateral or unilateral visual loss in adults in their 60's. MRI is now the preferred radiological diagnostic modality, and approximately one‐third of the cases show involvement of the optic tract or the contralateral nerve upon initial presentation. Positron emission tomography (PET‐CT) or MR spectroscopy imaging (MRSI) can sometimes aid in detecting the malignant nature of the lesion before histological confirmation.^18^, ^24^ Metabolic hyperactivity in PET‐CT or increased levels of choline compounds (Cho) along with decreased levels of N‐acetyl aspartate (NAA) in MRSI (Cho: NAA ratio >2) raise suspicion of malignancy in the lesion. However, primary optic GBM is frequently misdiagnosed and initially treated with corticosteroids.^15^, ^16^, ^25^, ^26^ Consequently, there is often a delay in obtaining a histological diagnosis, resulting in a postponement of appropriate treatment. We advocate for performing an open biopsy in cases of suspected space‐occupying lesions in the optic nerve or chiasma after ruling out common lesions such as lymphoma, sarcoidosis, and toxoplasmosis and when there is no response to short empirical therapy or persistence/deterioration of symptoms.

GBM generally carries a poor prognosis, with most patients dying within 24 months of establishing the diagnosis.^6^ Despite advancements in treatment modalities, the prognosis for GBM in the optic region remains unfavorable, and the 1‐year survival rate of 50% may be worse than that of GBM in other anatomical regions. However, comparing untreated cases with treated cases demonstrates an overall improvement in survival. Some clinicians have attempted novel therapy regimens. In this review, one case reported by Ashur‐Fabian, diagnosed with an open biopsy, was treated with radiochemotherapy and medically induced hypothyroidism. This particular case showed the longest survival of 54 months among those reviewed.^8^ In our case, surgical debulking (gross total resection) was performed, followed by radiochemotherapy, resulting in 18 months of progression‐free survival.

## 5 CONCLUSIONS

Primary glioblastoma of the optic nerve or chiasma is a rare condition that predominantly affects adults (mean age of presentation 61 years). This rarity often leads to misdiagnosis, mistreatment, and delays in confirming the histology. It is crucial to maintain a high level of suspicion for malignancy, particularly in adult patients with progressive symptoms, after excluding other potential differential diagnoses and observing nonresponsiveness to empirical treatment. Surgical debulking followed by radiochemotherapy has been shown to improve overall survival despite the associated morbidity, making it a justifiable therapeutic approach.

## AUTHOR CONTRIBUTIONS

**Ali Mulhem:** Conceptualization; data curation; formal analysis; investigation; methodology; software; visualization; writing – original draft; writing – review and editing.

## FUNDING INFORMATION

Not applicable.

## ETHICS STATEMENT

As this manuscript contains no identifiable information, the need for ethics approval was waived. Written informed consent was obtained from the patient.

## CONSENT

Written informed consent was obtained from the patient to publish this report in accordance with the journal's patient consent policy.

## Supporting information


Figures S1–S5.

## Data Availability

The author, AM, can provide the systematic review and meta‐analysis dataset upon request.
