# Supplementary material for: Primary glioblastoma multiform (GBM) of the optic nerve and chiasma: A case report and systematic review of the literature
Source: Clin Case Rep. 2024 Mar 20;12(3):e8636. doi: 10.1002/ccr3.8636 (PMC10954565; doi:10.1002/ccr3.8636)
Supplement: Supplementary file 1 — Figures S1–S5. [file CCR3-12-e8636-s001.zip › Figure captions.docx]

Figure S1: Visual field examination in Time 0

Figure S2: CT scan of the patient in parenchymal window, showing a suspected lesion on the left chiasma (red arrow)

Figure S3: MRI imaging of the patient in T1 sequence with no contrast, showing the lesion on the left chiasma isointense (red interrupted circle)

Figure S4: MRI imaging of the patient in T2 sequence, showing the lesion on the left chiasma relatively hyperintense (red interrupted circle)

Figure S5: Visual field examination in Time 1
