# Supplementary figures and images for: Primary glioblastoma multiform (GBM) of the optic nerve and chiasma: A case report and systematic review of the literature
Source: Clin Case Rep. 2024 Mar 20;12(3):e8636. doi: 10.1002/ccr3.8636 (PMC10954565; doi:10.1002/ccr3.8636)

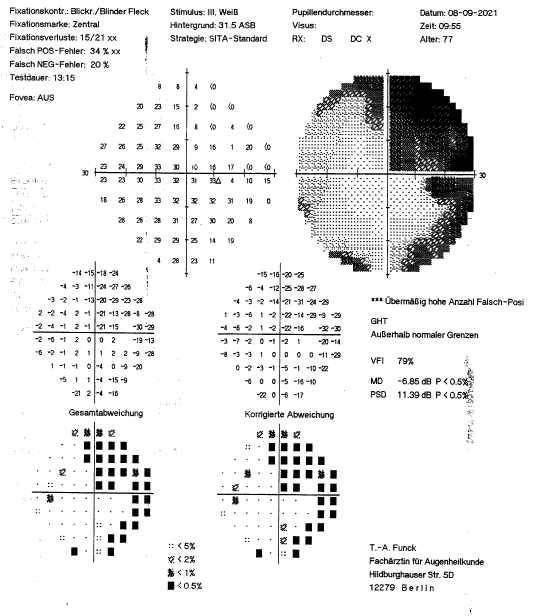

Supplement: Supplementary file 1 — Figures S1–S5. [file CCR3-12-e8636-s001.zip › ccr38636-sup-0001-FigureS1.PNG]

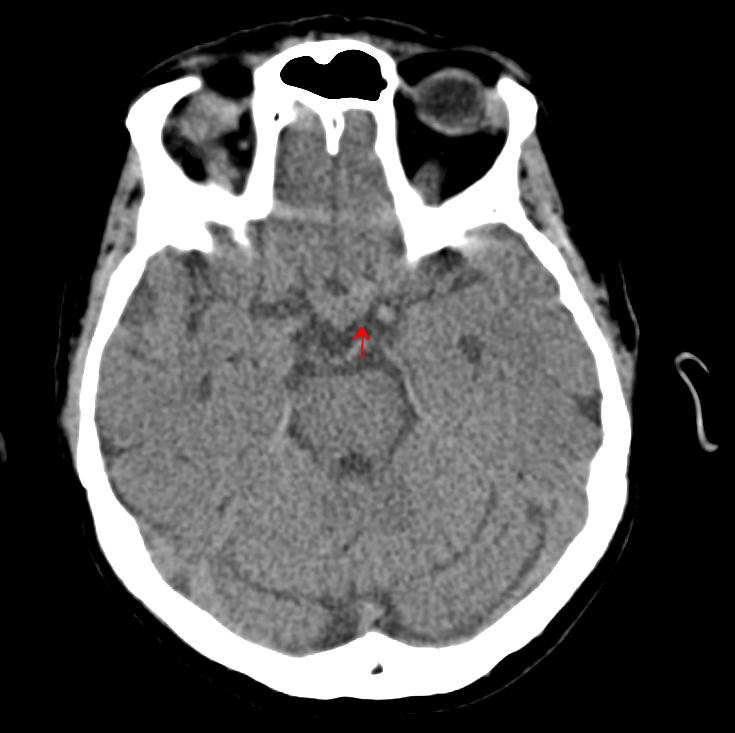

Supplement: Supplementary file 1 — Figures S1–S5. [file CCR3-12-e8636-s001.zip › ccr38636-sup-0002-FigureS2.PNG]

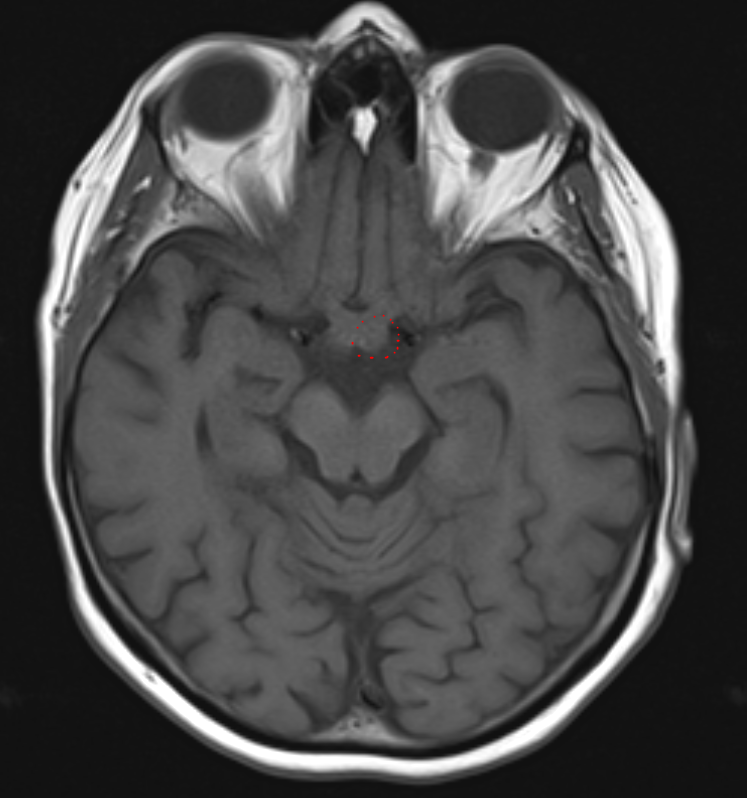

Supplement: Supplementary file 1 — Figures S1–S5. [file CCR3-12-e8636-s001.zip › ccr38636-sup-0003-FigureS3.PNG]

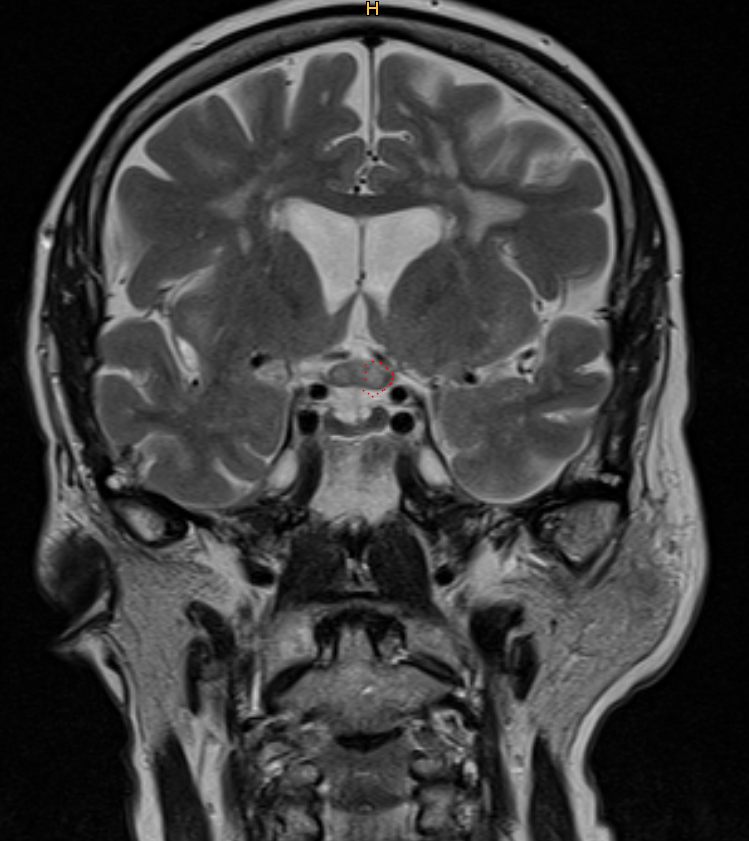

Supplement: Supplementary file 1 — Figures S1–S5. [file CCR3-12-e8636-s001.zip › ccr38636-sup-0004-FigureS4.PNG]

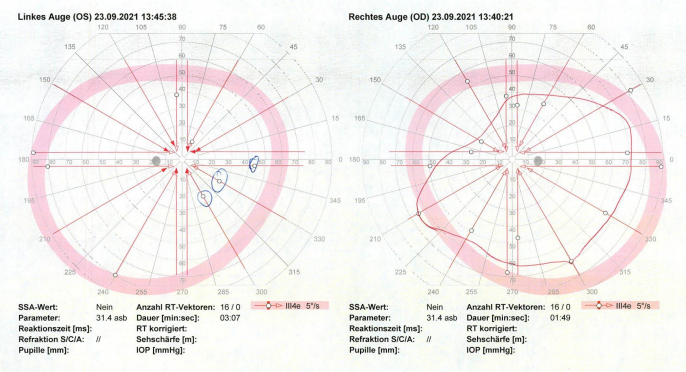

Supplement: Supplementary file 1 — Figures S1–S5. [file CCR3-12-e8636-s001.zip › ccr38636-sup-0005-FigureS5.PNG]
